# The Energetic Potential for Undiscovered Manganese Metabolisms in Nature

**DOI:** 10.3389/fmicb.2021.636145

**Published:** 2021-06-09

**Authors:** Douglas E. LaRowe, Harold K. Carlson, Jan P. Amend

**Affiliations:** ^1^Department of Earth Sciences, University of Southern California, Los Angeles, CA, United States; ^2^Department of Biological Sciences, University of Southern California, Los Angeles, CA, United States

**Keywords:** thermodynamics, bioenergetics, comproportionation, disproportionation, redox reactions

## Abstract

Microorganisms are found in nearly every surface and near-surface environment, where they gain energy by catalyzing reactions among a wide variety of chemical compounds. The discovery of new catabolic strategies and microbial habitats can therefore be guided by determining which redox reactions can supply energy under environmentally-relevant conditions. In this study, we have explored the thermodynamic potential of redox reactions involving manganese, one of the most abundant transition metals in the Earth’s crust. In particular, we have assessed the Gibbs energies of comproportionation and disproportionation reactions involving Mn^2+^ and several Mn-bearing oxide and oxyhydroxide minerals containing Mn in the +II, +III, and +IV oxidation states as a function of temperature (0–100°C) and pH (1–13). In addition, we also calculated the energetic potential of Mn^2+^ oxidation coupled to O_2_, NO_2_^–^, NO_3_^–^, and FeOOH. Results show that these reactions—none of which, except O_2_ + Mn^2+^, are known catabolisms—can provide energy to microorganisms, particularly at higher pH values and temperatures. Comproportionation between Mn^2+^ and pyrolusite, for example, can yield 10 s of kJ (mol Mn)^–1^. Disproportionation of Mn^3+^ can yield more than 100 kJ (mol Mn)^–1^ at conditions relevant to natural settings such as sediments, ferromanganese nodules and crusts, bioreactors and suboxic portions of the water column. Of the Mn^2+^ oxidation reactions, the one with nitrite as the electron acceptor is most energy yielding under most combinations of pH and temperature. We posit that several Mn redox reactions represent heretofore unknown microbial metabolisms.

## Introduction

Identifying the catabolic reactions that microorganisms catalyze in nature is critical to understanding the flows of energy and matter in ecosystems. Quantifying the amount of energy available from redox reactions among chemical species reveals which metabolisms could be operating. Gibbs energy calculations have been used in this way to survey the catabolic potential of a number of different ecosystems, such as terrestrial geothermal springs (Inskeep et al., 2005; Shock et al., 2005, 2010; Spear et al., 2005a, b; Windman et al., 2007; Vick et al., 2010; Berenguer, 2011; Cardace et al., 2015), deep-sea hydrothermal systems (Shock et al., 1995; McCollom and Shock, 1997; McCollom, 2000, 2007; Shock and Holland, 2004; Hentscher and Bach, 2012; Eecke et al., 2013; Dahle et al., 2015; Reed et al., 2015; McKay et al., 2016; Shibuya et al., 2016; Sylvan et al., 2017), shallow-sea hydrothermal systems (Amend et al., 2003, 2011; Rogers and Amend, 2005, 2006; Akerman et al., 2011; Boettger et al., 2013; LaRowe et al., 2014; Han and Perner, 2015; Price et al., 2015; Lu et al., 2020), marine sediments (LaRowe and Regnier, 2008; Schrum et al., 2009; Wang et al., 2010; LaRowe and Amend, 2014, 2015b; Teske et al., 2014; Kiel Reese et al., 2018), the terrestrial subsurface (Osburn et al., 2014), and marine basement rocks (Bach and Edwards, 2003; Edwards et al., 2005). These studies have shown that the energetics of redox reactions are fundamentally constrained by the nature of the compounds and the physiochemical properties of the environment, such as temperature, pressure, and chemical composition. In addition to revealing which catabolic strategies are potentially being used in an environment, Gibbs energy calculations reveal how much energy can be obtained from these reactions and therefore how many cells could be supported by them (Bach and Edwards, 2003; McCollom and Amend, 2005; Amend et al., 2013; LaRowe and Amend, 2014, 2015a,b, 2016; Bach, 2016; Bradley et al., 2018a, b, 2019, 2020).

**FIGURE 1:**
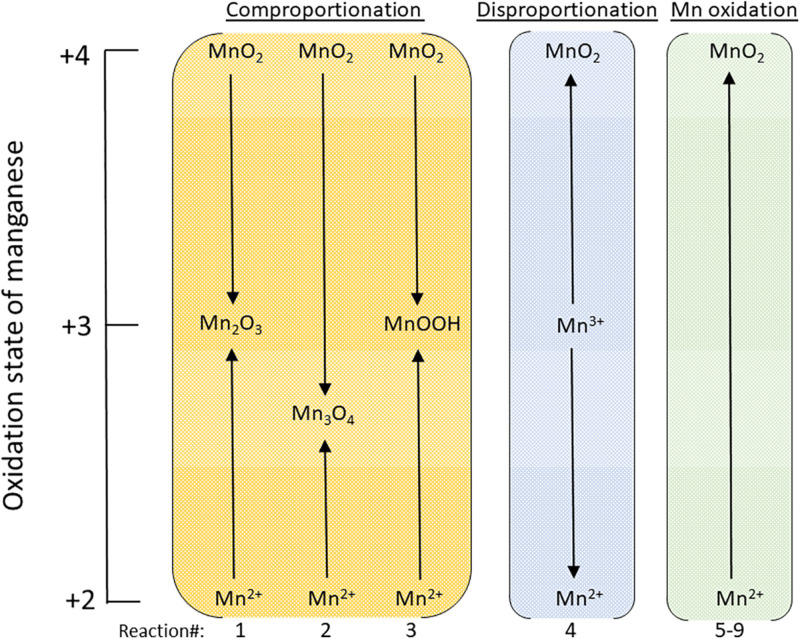
Schematic illustrating how the oxidation state of manganese changes for the comproportionation, disproportionation and Mn^2+^ oxidation reactions considered in this study. The reactions numbers at the bottom of the figure correspond to the reactions in Table 3. The oxidation state of hausmannite, Mn_3_O_4_, is shown as 2.67, the average for Mn in this phase: Mn^II^(Mn^III^)_2_O_4_.

Similar types of Gibbs energy calculations have been used to predict the existence of novel catabolic strategies that were later found in natural systems and built environments, such as anaerobic ammonia oxidation (anammox) (Broda, 1977; van de Graaf et al., 1995; Kuypers et al., 2003), the anaerobic oxidation of methane (AOM) (Barnes and Goldberg, 1976; Hinrichs et al., 1999; Boetius et al., 2000; Orphan et al., 2001) and complete ammonia oxidation (comammox) (Costa et al., 2006; Daims et al., 2015; van Kessel et al., 2015). Motivated by these successful thermodynamic prognostications, sulfur comproportionation, a heretofore undiscovered catabolic pathway, has recently been predicted to exist in ecosystems with acidic pH over a broad range of temperatures (Amend et al., 2020). These examples show that reactions among compounds formed from elements that have several oxidation states, such as N an S, are candidates for discovering novel catabolic strategies. Here, we have explored the energetic potential of a variety of undiscovered manganese-based microbial metabolisms including comproportionation, disproportionation, and oxidation by several electron acceptors including O_2_, NO_2_^–^, NO_3_^–^, and FeOOH, summarized schematically in Figure 1, as a function of temperature and pH. Redox reactions involving manganese-bearing compounds are likely candidates for novel catabolic strategies due to the ubiquity of Mn in Earth’s crust and the large number of microbial species that can enzymatically reduce and oxidize compounds containing it, as reviewed below. In this manuscript, we calculate the impact of temperature, pH and other compositional variables on the Gibbs energy of Mn redox reactions that could support microbial activities.

### Manganese in the Earth System

Mn oxides are found in ocean and lake sediments, ore deposits, soils, hydrothermal vents (Villalobos et al., 2003; Yang et al., 2018), interlayered with Fe-oxides that have recently become aerobic (Tazaki, 2000), caves (Northup et al., 2003) streams, and desert varnish (Tebo et al., 2004). Aqueous Mn(II), Mn^2+^, is common in suboxic and anoxic settings such as sediment pore water (Madison et al., 2013; Oldham et al., 2017b), stratified water bodies (Trouwborst et al., 2006; Yakushev et al., 2007, 2009; Dellwig et al., 2012; Oldham et al., 2015), ground water (Wasserman et al., 2006; de Meyer et al., 2017; McMahon et al., 2019) and drinking water systems (Cerrato et al., 2010). Mn(II) often coexists with birnessite (δ-MnO_2_) where redox conditions fluctuate, such as in ocean and lake sediments (Yang et al., 2018). The presence of aqueous Mn(III) in natural systems has recently become more appreciated, e.g., (Trouwborst et al., 2006; Madison et al., 2011, 2013; Oldham et al., 2015, 2017a,b), and in some settings, aqueous Mn(III) can constitute all or nearly all of the aqueous pool of dissolved Mn (Madison et al., 2011; Oldham et al., 2015, 2017b). Since aqueous Mn^3+^ rapidly disproportionates (Davison, 1993), aqueous Mn(III) is thought to be complexed to ligands that stabilize it, likely organic compounds (Heintze and Mann, 1947; Klewicki and Morgan, 1998; Parker et al., 2004; Duckworth and Sposito, 2005). Furthermore, trivalent Mn can also be stabilized in solid phase such as MnOOH through comproportionation reactions (Tu et al., 1994; Mandernack et al., 1995; Bargar et al., 2005; Elzinga, 2011, 2016; Elzinga and Kustka, 2015; Hinkle et al., 2016; Zhao et al., 2016; Wang et al., 2018), including during bacterial Mn(IV) reduction (Johnson et al., 2016). Finally, it is noteworthy, that unlike Fe in many settings, dissolved Mn passes through a 0.02 micron filter, indicating that it is actually an aqueous species, not part of a colloid (Oldham et al., 2017b). See Table 1 for a selection of environments in which Mn concentrations in natural settings have been reported.

**TABLE 1:** Concentration of Mn in selected environmental settings.

Environment	Species or phase	Concentration	References
Black Sea water column (various depths)	Dissolved Mn	0.49–2.15 μmol L^–1^	Clement et al., 2009
	Particulate Mn-oxides	0.01–1.4 μmol L^–1^	
Chesapeake Bay water column	Particulate Mn-oxide	0–4.89 μmol L^–1^	Oldham et al., 2015
	Mn(II),aq	0.59–8.04 μmol L^–1^	
	Mn(III),aq	0–6.98 μmol L^–1^	
North Atlantic Water Column	Particulate Mn oxides	0.19–3.5 nmol L^–1^	Jones et al., 2020
	Mn(III)-ligand, aq	0.01–0.83 nmol L^–1^	
	Mn(II), aq	0.5–25 nmol L^–1^	
Oneida Lake bottom water	Mn(II), aq	0.48–3.3 μmolL^–1^	Chapnick et al., 1982
Mouth/Lower St. Lawrence Estuary sediment	Mn Oxide	0 –130 μmol g-1	Madison et al., 2013
	Mn(II), aq	0–200 μmol L^–1^	
	Mn(III), aq	0–70 μmol L^–1^	
Amazon fan sediment	Mn(II)	3.2 g Mn (kg sediment)^–1^	Kasten et al., 1998
Fe-Mn nodule-rich marine sediment pore water	Mn(II)	0–38 μmol L^–1^	de Lange et al., 1992
Various Fe-Mn Nodules	Mn	15.9–34.2 weight %	Hein, 2013
Swiss lake sediment porewater	Shallow water Mn, aq	10–30 μmol L^–1^	Schaller and Wehrli, 1996
	Deep water Mn, aq	110–350 μmol L^–1^	
Groundwater in China	Mn, aq	0–62.1 μmol L^–1^	Hou et al., 2020
Groundwater in Scotland	Mn, aq	0–35 μmol L^–1^	Homoncik et al., 2010
Groundwater in the United States	Mn,aq	0–20,630 μmol L^–1^	McMahon et al., 2019
Drinking water, rural Bangladesh	Mn, aq	2-100 μmol L^–1^	Akter et al., 2016
Hydrothermal vent plumes, Juan de Fuca Ridge	Dissolved Mn	0–∼600 nmol L^–1^	Chin et al., 1994
Hydrothermal plume and surrounding bottom water, Galapagos Rift	Total dissolvable Mn	0.41–24 μg per kg	Klinkhammer et al., 1977
San Clemente Basin sediment (near cold seep)	Dissolved Mn	0–∼600 μmol L^–1^	McQuay et al., 2008
Atlantic pelagic sediment pore water	Mn(II)	0–100 μmol L^–1^	Froelich et al., 1979
River Leie sediment pore water, Menen Belgium	Total Dissolved Mn	3.77–39.1 μmol L^–1^	Gao et al., 2007
Zambezi fan sediment	Mn(II)	∼2–12 μmol L^–1^	März et al., 2008
	Solid Mn	∼0.3–∼0.4 g/kg	
German tidal wetlands (median)	Dissolved Mn	8.4 μmol L^–1^	Hamer et al., 2020

### Microbial Processing of Manganese

Microorganisms can reduce and oxidize Mn compounds to gain energy. Though no obligate Mn-reducers are known, the biological reduction of Mn-oxides to Mn^2+^ has been shown to occur in a number of environments (Burdige and Nealson, 1985; Lovley and Phillips, 1988; Myers and Nealson, 1988; Tebo et al., 1991; Burdige et al., 1992; Burdige, 1993; Gounot, 1994; Henkel et al., 2019). Microbial Mn(II) oxidation is phylogenetically widespread, occurring in bacteria, archaea, and eukarya (Hansel, 2017), and the enzymes associated with this process are diverse (Wright et al., 2018). A community of microorganisms has even been shown to photooxidize Mn^2+^ under anoxic conditions (Daye et al., 2019). Taken together, Mn^2+^ oxidation is thought to be responsible—directly or by environmental modification—for the formation of the majority of Mn oxides in nature (Tebo et al., 2004). Although this process has been well-studied, e.g., (Nealson et al., 1988; Tebo et al., 2004; Hansel, 2017), it was only recently shown that a microorganism can catalyze Mn^2+^ oxidation to gain energy (Yu and Leadbetter, 2020). It has also been demonstrated that microorganisms can reduce aqueous ligand-bound Mn(III) (Kostka et al., 1995; Szeinbaum et al., 2014, 2017, 2020) and solid-phase Mn(III), in the form of manganite (MnOOH) (Larsen et al., 1998; Fredrickson et al., 2002), to provide energy for microorganisms.

Mn oxidation and reduction are known to take place simultaneously in the same system, and there are isolates known that can both reduce and oxidize Mn, e.g., *Lysinibacillus fusiformis*, *Bacillus pumilus*, and *B. cereus* (Cerrato et al., 2010). Phylogenetic studies of iron-manganese nodules on the seafloor have shown that the associated microbial communities are significantly distinct from those in surrounding sediments and that the interior communities are different from the exteriors of these nodules, suggesting that more diversity on the interior could indicate Mn cycling (Tully and Heidelberg, 2013). A metagenomic study on ferromanganese crusts on Takuyo-Daigo Seamount found putative genes for dissolution and precipitation of Mn, including protein-coding DNA sequences similar to outer-membrane *c*-type cytochromes that *Shewanella* spp. use to reduce Mn(IV) and protein-coding DNA sequences similar to Mn oxidases such as MopA and multicopper oxidase sequences (Kato et al., 2019). In shallower ocean settings, Mn^2+^ can be found with layered Mn-oxides when Mn^2+^ diffuses upward in sediments into oxic zones (Yang et al., 2018). Microfossil evidence in ferromanganese nodules and crusts support the notion that microbial activity is responsible for concentrating Mn in nodules and crusts from seawater (Jiang et al., 2019), where Mn concentration is typically 0.1–0.15 nm (van Hulten et al., 2017). Similarly, nodules from the NE Equatorial Pacific were revealed to have connected pore space and molecular data showed that the microbial community was dominated by nodule-specific Mn(IV)-reducing and Mn(II)-oxidizing bacteria that were not found in the surrounding environment (Blöthe et al., 2015).

## Materials and Methods

Values of overall Gibbs energies at the conditions of interest, Δ*G*_*r*_, are calculated using:

(1) $$\Delta G_{r}=\Delta G_{r}^{0}+RT\text{ln}Q_{r}$$

where $\Delta G_{r}^{0}$ and *Q*_*r*_ refer to the standard molal Gibbs energy and the reaction quotient of the indicated reaction, respectively, *R* represents the gas constant, and *T* denotes temperature in Kelvin. Values of $\Delta G_{r}^{0}$ were calculated using the revised-HKF equations of state (Helgeson et al., 1981; Tanger and Helgeson, 1988; Shock et al., 1992), the SUPCRT92 software package (Johnson et al., 1992), and thermodynamic data taken from a number of sources (Robie and Bethke, 1963; Bricker, 1965; Helgeson et al., 1978; Hem et al., 1982; Robie and Hemingway, 1985; Shock and Helgeson, 1988; Shock et al., 1997; Chase, 1998; Senoh et al., 1998; Schulte et al., 2001; Snow et al., 2013; LaRowe and Amend, 2014; see Table 2). Values of *Q*_*r*_ are calculated using:

(2) $$Q_{r}=\prod_{i}a_{i}^{\nu_{i}},$$

**TABLE 2:** Summary of the standard molar thermodynamic properties at 25°C and 1 bar and heat capacity power function coefficients (*a*, *b*, and *c*) for selected Mn-bearing minerals.

Compound	Formula	Δ*G*_*f*_^0a^	Δ*H*_*f*_^0a^	*S*^0b^	*V*^0c^	*a*^*d*^	*b*^*e*^	*c*^*f*^	T_*max*_/T_*range*_ (K)
Pyrolusite	MnO_2_	−465,000^*g*^	−520,000^*h*^	52.75^*h*^	16.61^*i*^	51.47^*j*^	42.78^*j*^	−8.368^*j*^	1,000
Bixbyite	Mn_2_O_3_	−882,100^*g*^	−959,000^*h*^	113.7^*h*^	31.38^*i*^	−67.51^*j*^	521.7^*j*^	19.36^*j*^	240–300
						217.2^*j*^	−355^*j*^	0j	300–325
						101.4^*j*^	36.59^*j*^	−11^*j*^	325–1,400
Hausmannite	Mn_3_O_4_	−1,279,000^*g*^	−1,384,500^*h*^	165.60^*h*^	46.96^*i*^	149.5^*j*^	52.75^*j*^	−20.02^*j*^	270–1,100
Feitknechtite	β-MnOOH	−543,100^*k*^							
Nsutite	γ-MnO_2_	−456,500^*l*^							
Manganite	γ-MnOOH	−557,700^*l*^							
Birnessite	δ-MnO_2_	−453,100^*l*^							
Pyrochroite	Mn(OH)_2_	−615,630^*l*^							
Amorphous Mn(OH)_2_	Mn(OH)_2_	−615,000^*m*^							

*^a^J mol^–1^; ^b^J K^–1^ mol^–1^; ^c^cm^3^ mol^–1^; ^d^J mol^–1^; ^e^10^3^ J K^–2^ mol^–1^; ^f^10^–5^ J K mol^–1^; ^g^calculated from ΔH^0^ and S^0^ and S^0^ of the elements taken from Chase (1998); ^h^Robie and Hemingway (1985); ^i^Robie and Bethke (1963); ^j^calculated by regressing isobaric heat capacity data as a function of temperature from h using the Maier–Kelly equation (see section “Materials and Methods”); ^k^Hem et al. (1982); ^l^Bricker (1965); ^m^Senoh et al. (1998). T_max_/T_range_ refers to, respectively, the maximum temperature and temperature range to which the thermodynamic data are valid.*

where *a*_*i*_ stands for the activity of the *i*th species and *v*_*i*_ corresponds to the stoichiometric coefficient of the *i*th species in the reaction of interest. Negative values of Δ*G*_*r*_ are said to be exergonic and positive values are endergonic; Δ*G*_*r*_ = 0 defines equilibrium. Because standard states in thermodynamics specify a composition and state of aggregation (Amend and LaRowe, 2019; LaRowe and Amend, 2020) values of *Q*_*r*_ must be calculated to take into account how environmental conditions impact Gibbs energy calculations. In this study we use the classical chemical-thermodynamic standard state in which the activities of pure liquids and solids are taken to be 1 as are those for aqueous species in a hypothetical 1 molal solution referenced to infinite dilution at any temperature or pressure. Additional information detailing how the Gibbs energy calculations were carried out can be seen in the Supplementary Materials.

Activities are related to concentration, *C*, by

(3) $$a_{i}=\gamma_{i}\left(\frac{C_{i}}{C_{i}^{\Theta }}\right)$$

where γ*_*i*_* and *C*_*i*_ stand for the individual activity coefficient and concentration of the *i*th species, respectively, and C_*i*_^θ^ refers to the concentration of the *i*th species under standard state conditions, which is taken to be equal to one molal referenced to infinite dilution. Values of γ*_*i*_* can be computed using an extended version of the Debye–Hückel equation (Helgeson, 1969). Values of γ*_*i*_* vary, mostly, as a function of temperature, ionic strength and charge. For reference, γ*_*i*_* for Mn^2+^ in seawater at 25°C and 1 bar is 0.16. Therefore, *a*_Mn_^2+^ = 10^–6^ corresponds to a concentration of 6.25 μmol (kg H_2_O)^–1^ under these conditions. For other temperatures, charge states and ionic strengths, see Amend and LaRowe (2019) for values of γ*_*i*_*.

The calculations summarized in the figures discussed below have been carried out over a range of plausible natural conditions (see Table 1). We have focused on pH, – $\log a_{{\text{H}}^{+}}$, because it tends to be a master variable in natural settings and it can vary by many orders of magnitude, thereby significantly altering the energetic potential of a reaction that has hydrogen ions in it. The activities of the other aqueous species, O_2_, NO_2_^–^, NO_3_^–^, N_2_, NH_4_^+^, Mn^2+^, and Mn^3+^, tend to vary less than H^+^. Their activities are meant to be representative of common natural settings. To illustrate the impact of variable Mn^2+^ activities, we have also calculated the Gibbs energies of two reactions, those with the largest and smallest stoichiometric numbers for Mn^2+^, as a function of *a*_Mn_^2+^. The Gibbs energies of Mn^2+^ oxidation by O_2_ is included in this analysis as a basis of comparison for the other Mn^2+^ oxidation reactions as well as because it has only recently been shown to support the energetic needs of an organism under one set of compositional conditions (Yu and Leadbetter, 2020).

Although the thermodynamic data required to calculate the Gibbs energies of Mn-oxides as a function of temperature have been available for decades, they have not been presented in a format amenable to commonly used thermodynamic software such as SUPCRT, OBIGT, EQ3/6, and CHNOSZ [see Dick (2019) and chnosz.net for a discussion of thermodynamic databases]. Consequently, these data are presented along with the parameters used to calculate thermodynamic variables as a function of temperature, as regressed using the Maier–Kelly equation (Maier and Kelley, 1932), in Table 2 (i.e., the *a*, *b*, and *c* parameters). The thermodynamic properties of pyrolusite (MnO_2_) are used in the Gibbs energy calculations in place of the more commonly abundant birnessite (δ-MnO_2_) because the thermodynamic properties for pyrolusite are known as a function of temperature and those for birnessite are not. As can be seen in Table 2, there is a 2.6% difference in the Gibbs energies of formation for these two phases at 25°C and 1 bar.

## Results

**FIGURE 2:**
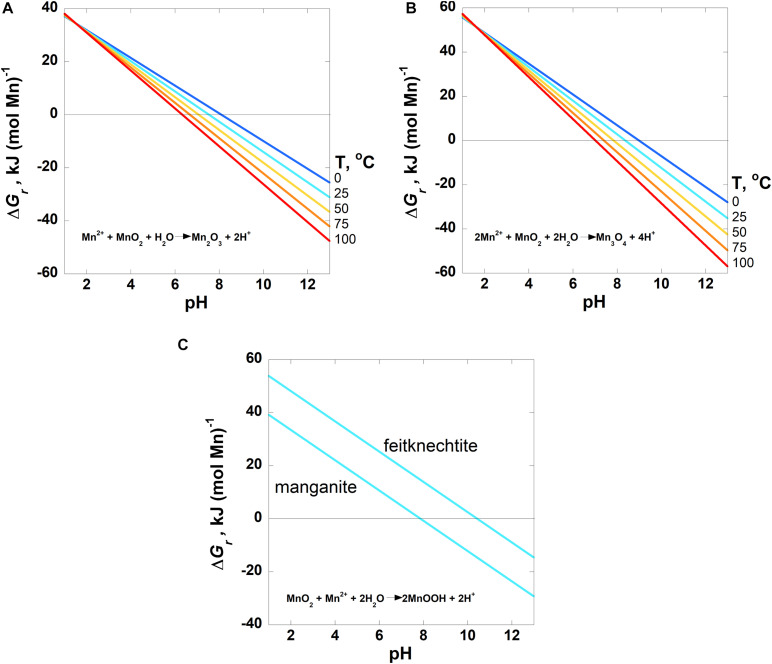
Overall Gibbs energies, Δ*G*_*r*_, of the comproportionation reactions listed in Table 3 (Reactions 1–3) as a function of pH from 0 to 100°C for **(A)** bixbyite and **(B)** hausmannite formation and **(C)** at 25°C for MnOOH, manganite and feitknechtite, formation. For all three reactions, the activity of Mn^2+^ = 10^–6^. Activities of H_2_O and all solid phases are taken to be 1. The horizontal line in each panel designates where Δ*G_*r*_* = 0; Gibbs energies below this line are exergonic.

**FIGURE 3:**
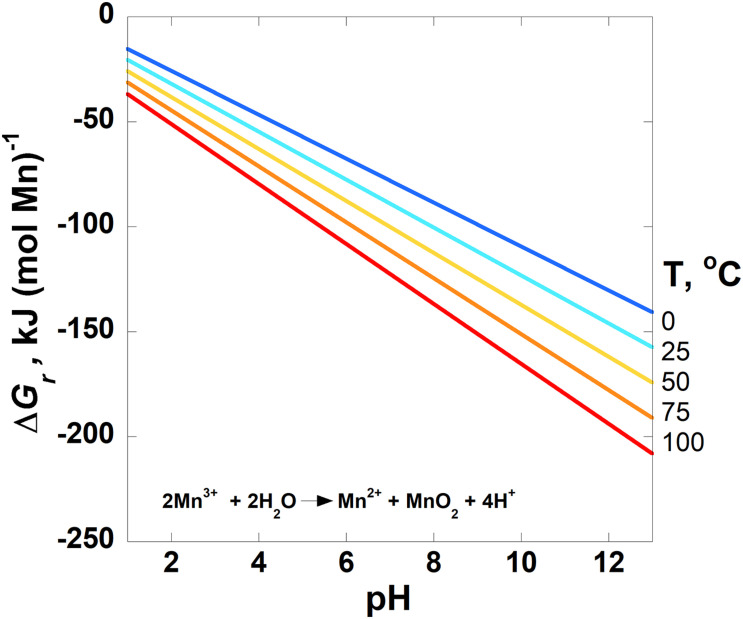
Overall Gibbs energies, Δ*G*_*r*_, of the disproportionation reaction given in Table 3 (Reaction 4) as a function of pH from 0 to 100°C for pyrolusite formation. The activities of Mn^2+^ and Mn^3+^ are set to 10^–6^. Activities of H_2_O and all solid phases are taken to be 1. The horizontal line designates where Δ*G_*r*_* = 0; Gibbs energies below this line are exergonic.

**FIGURE 4:**
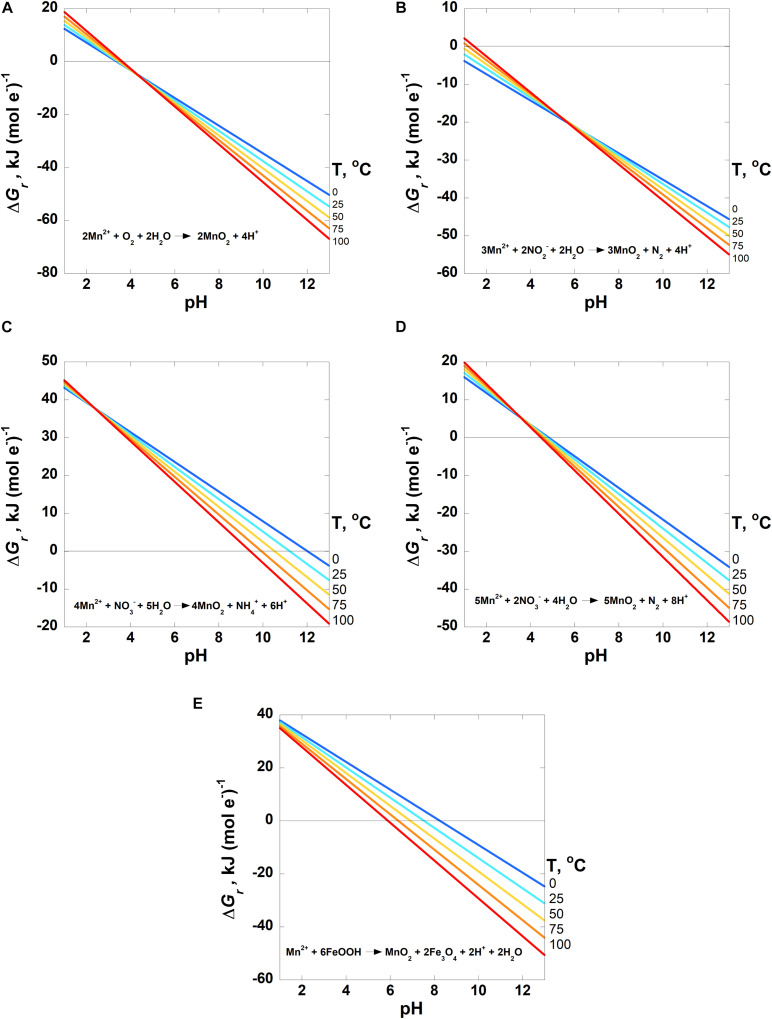
Overall Gibbs energies, Δ*G*_*r*_, of the Mn^2+^ oxidation reactions listed in Table 3 [Reactions 5–9 in panels **(A–E)**, respectively] as a function of pH from 0 to 100°C. Activities of H_2_O and all solid phases are taken to be 1 and the activity of Mn^2+^ = 10^–6^. The activities of the other species are **(A)** O_2_ = 10^–4^; **(B)** NO_2_^–^ = 10^–7^ and N_2_ = 10^–4^; **(C)** NO_3_^–^ = 10^–5^ and NH_4_^+^ = 10^–5^; **(D)** NO_3_^–^ = 10^–5^ and N_2_ = 10^–4^. The horizontal line in each panel designates where Δ*G_*r*_* = 0; Gibbs energies below this line are exergonic.

Values of the overall Gibbs energies, Δ*G*_*r*_, of the reactions listed in Table 3, hereafter referred to by the reaction numbers in this table only, are shown as a function of pH in Figures 2–4 from 0 to 100°C with the exception of the comproportionation reactions involving both MnOOH phases (Figure 2C, Reaction 3), which are shown only at 25°C, the extent of the thermodynamic data for these phases. Since the hydrogen ion is on the right side of all of the reactions considered in this communication, values of Δ*G*_*r*_ become more negative and thus more favorable as pH increases. In general, Mn reactions are more exergonic at higher temperatures than lower ones, particularly as pH values increase. The activities of several species are fixed at the values noted in each figure caption to reduce the number of figures to a comprehensible total. The impact of varying these activities on Gibbs energies of reactions is proportional to the stoichiometric coefficients in front of them, as per Equation 2. Values of Δ*G*_*r*_ for the Mn^2+^ oxidation reactions are reported in units of kJ (mol e^–^)^–1^ to facilitate comparison amongst these reactions as well as other such reactions reported in the literature that also use these units (see section “Introduction”). It is clear how many electrons are transferred between reactants and products in these reactions [e.g., Mn^2+^ oxidation to MnO_2_ represents a two electron transfer; Mn(II) becomes Mn(IV)]. However, units of kJ (mol Mn)^–1^ are used for the comproportionation and disproportionation reactions because the average oxidation state of Mn is the same on both sides of these reactions, obfuscating how the number of electrons transferred in the process should be counted. This follows how the Gibbs energies were reported for a number of fermentation (i.e., disproportionation) reactions (LaRowe and Amend, 2019).

**TABLE 3:** Manganese catabolic reactions considered in this study.

**Comproportionation reactions**
1 MnO_2_ + Mn^2+^ + H_2_O → Mn_2_O_3_ + 2H^+^
2 MnO_2_ + 2Mn^2+^ + 2H_2_O → Mn_3_O_4_ + 4H^+^
3 MnO_2_ + Mn^2+^ + 2H_2_O → 2MnOOH + 2H^+^
**Disproportionation reaction**
4 2Mn^3+^ + 2H_2_O → MnO_2_ + Mn^2+^ + 4H^+^
**Mn oxidation reactions**
5 2Mn^2+^ + O_2(aq)_ +2H_2_O → 2MnO_2_ + 4H^+^
6 3Mn^2+^ + $2{\text{NO}}_{2}^{-}$ +2H_2_O → 3MnO_2_ +N_2(aq)_ + 4H^+^
7 4Mn^2+^ + ${\text{NO}}_{3}^{-}$ +5H_2_O → 4MnO_2_ +$N{\text{H}}_{4}^{+}$ + 6H^+^
8 5Mn^2+^ + $2{\text{NO}}_{3}^{-}$ +4H_2_O → 5MnO_2_ + N_2(aq)_ + 8H^+^
9 Mn^2+^ + 6FeOOH → MnO_2_ + 2Fe_3_O_4_ + 2H_2_O + 2H^+^

*The following chemical formulas refer to the indicated crystalline Mn phases: MnO_2_ (pyrolusite), Mn_2_O_3_ (bixbyite), Mn_3_O_4_ (hausmannite), MnOOH (manganite and feitknechtite), FeOOH (2-line ferrihydrite), and Fe_3_O_4_ (magnetite).*

**FIGURE 5:**
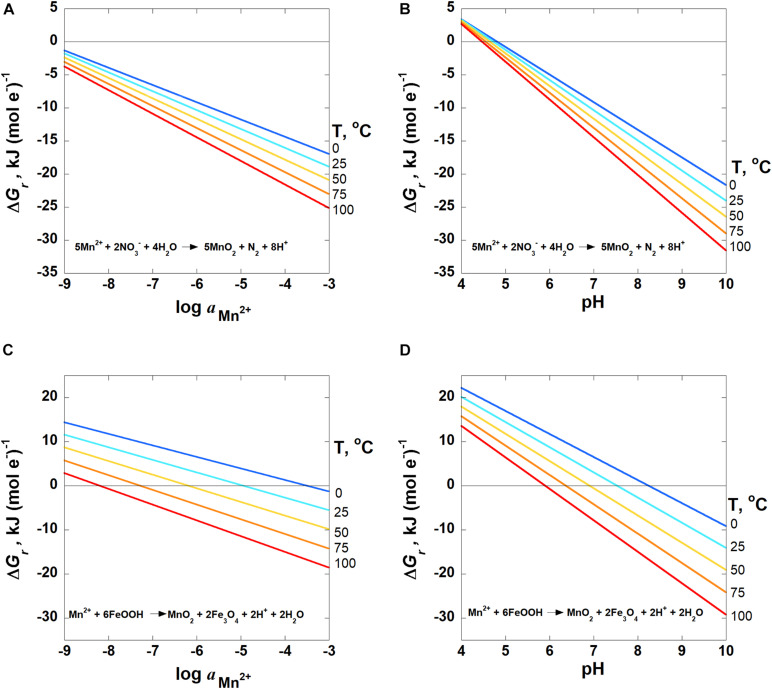
Overall Gibbs energies, Δ*G*_*r*_, of Reactions **(A,B)** 8 and **(C,D)** 9 as a function of **(A,C)***log*⁡*a*_Mn_^2+^ at pH = 7 and **(B,D)** pH for *a*_Mn_^2+^ = 10^−6^ from 0 to 100°C. Activities of H_2_O and all solid phases are taken to be 1. The activities of N_2_ and NO_3_^–^ are 10^–4^ and 10^–5^, respectively. The horizontal line in each panel designates where Δ*G_*r*_* = 0; Gibbs energies below this line are exergonic.

The impact of Mn^2+^ activities on the Gibbs energies of Reactions 8 and 9 are plotted in Figures 5A,C from 0 to 100°C at pH 7. Since Mn^2+^ is on the left-hand side of these reactions, increasing activities of Mn^2+^ results in lower values of Δ*G*_*r*_ for all temperatures. In the case of nitrate reduction, Reaction 8, Gibbs energies at 25°C decrease from −1.8 kJ (mol e^–^)^–1^ at *a*_Mn_^2+^ = 10^–9^ to −18.9 kJ (mol e^–^)^–1^ at *a*_Mn_^2+^ = 10^–3^. By comparison, Δ*G*_*r*_ for Reaction 9, ferrihydrite reduction, drops from 11.6 to −5.5 kJ (mol e^–^)^–1^ over the same *a*_Mn_^2+^ range at 25°C. The impact of Mn activities is only shown for two reactions to illustrate the relative impact of this variable on reaction energetics. The particular reactions chosen are those that have the largest and smallest stoichiometric numbers for Mn^2+^, and therefore values of Δ*G*_*r*_ that are the most and least sensitive to Mn^2+^ activities (see Equations 1, 2).

The Gibbs energies of three comproportionation reactions among pyrolusite and Mn^2+^, forming bixbyite (Mn_2_O_3_), hausmannite (Mn_3_O_4_) and two manganese oxyhydroxide phases (MnOOH–manganite and feitknechtite), were considered in this study (see Reactions 1–3; Figure 2A) along with one disproportionation reaction (Reaction 4; Figure 3). The results are normalized to units of kJ (mol Mn)^–1^. The comproportionation reactions forming bixbyite and hausmannite are exergonic at ∼pH > 6 at 100°C. Higher pHs are necessary at lower temperatures for these reactions to be favored: pH ∼7 at 50°C and pH ∼8 at 0°C. The comproportionation reactions forming manganite and feitknechtite, shown in Figure 2C, are exergonic above pH 8 and 10, respectively, at 25°C. In contrast to these comproportionation reactions, the disproportionation of Mn^3+^ to Mn^2+^ and pyrolusite (Reaction 4; Figure 3), is exergonic from 0–100°C throughout the pH range considered. At all pH values, Gibbs energies are lower (more favorable) for Reaction 4 as temperatures increase. In addition, the values of Δ*G*_*r*_ for this reaction are three to six times more exergonic than the disproportionation reactions.

The energetic potentials of Mn^2+^ oxidation by O_2(aq)_, NO_2_^–^, NO_3_^–^ and 2-line ferrihydrite (FeOOH) (Reactions 5–9) are shown in the panels in Figure 4 as a function of temperature and pH for the indicated activities of the aqueous species in each reaction. Slightly different from Reactions (1–4) in Figures 2, 3, the results of these reactions are shown per mole of electron transferred. The reduction of oxygen (Reaction 5, Figure 4A) is exergonic at all temperatures for pH values above ∼3.7, varying slightly with temperature. Values of Δ*G*_*r*_ for Reaction 6, in which nitrite is the oxidant, are exergonic throughout nearly the entire pH and temperature range considered, with the only exceptions being at 75 and 100°C below pH 2 (Figure 4B). Figures 4C,D both show the Gibbs energies of Mn^2+^ oxidation with nitrate (Reactions 7 and 8), but differ in the oxidation state of the nitrogen product species (NH_4_^+^ and N_2_, respectively). The major difference between these reactions is that the complete reduction of NO_3_^–^ to NH_4_^+^ is less exergonic per electron transferred than the partial reduction to N_2_. Reaction 8 (N_2_ formation) becomes exergonic from about pH 6–7, depending on temperature, while Reaction 7 (NH_4_^+^ formation) does not become favorable until about pH 9.5–12, from 100 to 0°C. Finally, values of Δ*G*_*r*_ for the oxidation of Mn^2+^ coupled to the reduction of FeOOH (Reaction 9; Figure 4E) become exergonic over a pH range of 6–8, depending on temperature.

**FIGURE 6:**
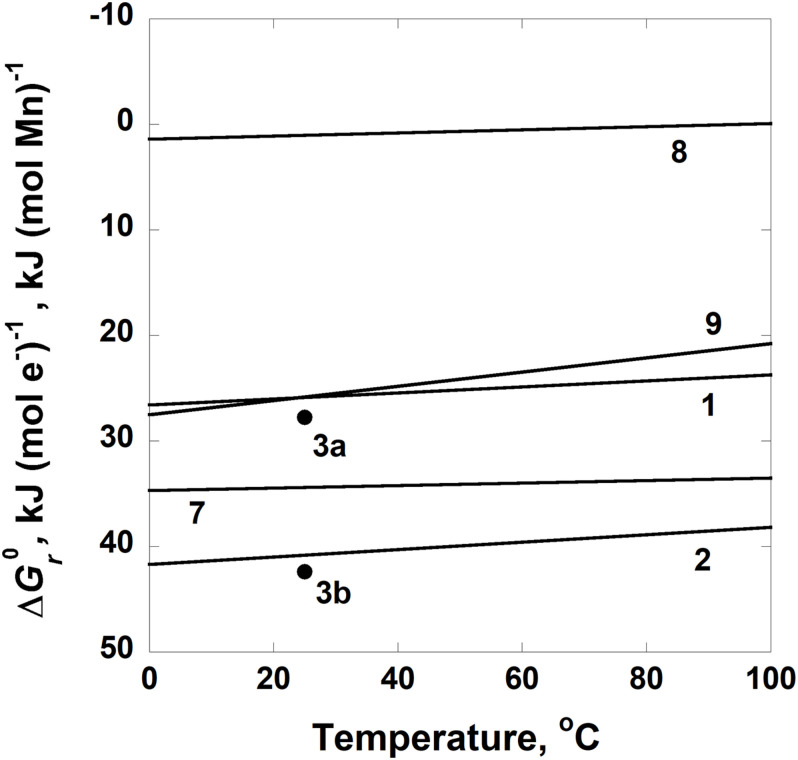
Standard state Gibbs energies, Δ$G_{r}^{0}$, of Reactions 1–3 and 7–9 in Table 3 as a function of temperature. Units of kJ (mol Mn)^–1^ are given for Reactions 1–3 and kJ (mol e^–^)^–1^ for Reactions 7–9. The solid circles labeled 3a and 3b refer to values of Δ$G_{r}^{0}$ for Reaction 4 at 25°C where manganite and feitknechtite, respectively, are the phases of MnOOH.

The standard state Gibbs energies, Δ$G_{r}^{0}$, of Reactions 1, 2, 3, 7, 8, and 9 are shown as a function of temperature in Figure 6. This subset of reactions is illustrated because Δ$G_{r}^{0}>0$ for all of them at all temperatures except above 95°C for Reaction 8. In fact, values of the standard state Gibbs energies for each of these reactions, except Reaction 8, are greater than 20 kJ (mol e^–^)^–1^ or (mol Mn)^–1^. Both sets of units appear on the y-axis since the comproportionation and disproportionation reactions are normalized per mole of Mn and the oxidation reactions are normalized per mole of electron transferred.

Four other oxidants were also considered in possible oxidation reactions of Mn^2+^ to pyrolusite (CO to CH_4_; NO_3_^–^ to NO_2_^–^; magnetite to Fe^2+^; ferrihydrite to Fe^2+^), but none of these reactions was exergonic over a broad range of temperature, pH, and other compositional conditions (not shown).

## Discussion

The calculations presented above demonstrate that comproportionation and disproportion reactions involving Mn species, as well as Mn^2+^ oxidation with various electron acceptors, could provide energy for microorganisms. However, these reactions can only be catalyzed by organisms in environments where the composition and temperature allow it. The impact of taking into account non-standard state activities of reactants and products on energy yields is clearly shown in Figures 2–6, where standard state and overall Gibbs energies of reactions are compared. Note that values of Δ$G_{r}^{0}$ are positive throughout nearly the entire range of temperatures considered, but those of Δ*G_r*, which take into account non-standard state compositions, can be negative (i.e., exergonic). Our results illustrate the importance of pH in determining the exergonicity of reactions involving Mn: with the exception of the Mn^3+^ disproportionation reaction (Reaction 4), all of the reactions considered in this study are not thermodynamically favored at low pH. It should be noted that just because a given reaction is exergonic under a particular set of environmental conditions, this does not necessarily mean that organisms will catalyze it. The thermodynamic favorability of reactions indicated by Gibbs energy is a statement of the possible—it quantifies the tendency of a chemical reaction to proceed in a particular direction. Gibbs energy calculations do not reveal the path of a process or information about intermediate species or reactions that might be occurring. However, Δ*G*_*r*_ can still quantify the potential for complex, multi-organism processes such as AOM. The microbial coupling of methane oxidation to sulfate reduction was predicted to exist thermodynamically before it was demonstrated to occur in nature. A large body of research has since shown that AOM is catalyzed by a consortia of microorganisms through a rather complex series of steps that are yet to be fully understood [see Knittel et al. (2019) for a review]. However, because the overall process can be represented by a chemical reaction that accurately describes how chemical species are transformed, the Gibbs energy of the AOM reaction can be used to quantify the amount of energy associated with the overall change. In a similar manner, the Mn reactions considered in this study might not capture the complexity of how organisms in nature might take advantage of them for energy, but as long as the overall process corresponds to the observed mass transfer associated with this reaction, then the Gibbs energies reported in this study are a valid prediction of possible catabolisms and provide a theoretical basis for future research.

Values of Gibbs energies for the reactions shown in Table 3 are more sensitive to pH than the activity of Mn^2+^. This is because the stoichiometric numbers in front of H^+^ are larger than those in front of Mn^2+^ for any given reaction. The quantitative difference of pH vs. Mn^2+^ activity, *a*_Mn_^2+^, on values of Δ*G*_*r*_ are shown in Figure 5. As noted above, Figures 5A,C show Δ*G*_*r*_ for Reactions 8 and 9 as a function of *a*_Mn_^2+^ at pH 7. Figures 5B,D are rescaled versions of Figures 4D–E, illustrating Gibbs energies of Reactions 8 and 9 as a function of pH at a *a*_Mn_^2+^ = 10^6^. It can be seen in Figure 5 that the slopes of the lines depicting Δ*G*_*r*_ as a function of pH are steeper and cover a broader range of values than those plotted as a function of *a*_Mn_^2+^ for the same reaction. For example, Gibbs energies at 25°C for nitrate reduction, Reaction 8, change from −1.8 kJ (mol e^–^)^–1^ at *a*_Mn_^2+^ = 10^−9^ to −18.9 kJ (mol e^–^)^–1^ at *a*_Mn_^2+^ = 10^−3^ (Figure 5A). For the same order of magnitude change in pH, values of Δ*G*_*r*_ for the same reaction change from 3.4 kJ (mol e^–^)^–1^ at pH 4 to −24.0 kJ (mol e^–^)^–1^ at pH 10 (Figure 5C). Similarly, Δ*G*_*r*_ for Reaction 9, ferrihydrite reduction, drops from 11.6 kJ (mol e^–^)^–1^ to −5.5 (mol e^–^)^–1^ over the same six-order of magnitude Mn^2+^ range at 25°C, and from 20.2 kJ (mol e^–^)^–1^ to −14.1 kJ (mol e^–^)^–1^ from pH 4–10.

The results presented above also illustrate that substantial differences in reaction energetics can correspond to seemingly subtle differences in the identity of reaction products. For example, values of Δ*G*_*r*_ for Reaction 3 differ by ∼15 kJ (mol Mn)^–1^ depending on whether manganite or feitknechtite (both MnOOH) are the reaction product, a point that has been made for analogous Fe-oxyhydroxide species (LaRowe and Amend, 2014). Similarly, we show that the energetics of oxidation of Mn^2+^ by NO_3_^–^, Reactions 7 and 8, depend dramatically on the identity of the product species formed. As shown in Figures 4C,D, when N_2_ is the product N species rather than NH_4_^+^, the values of Δ*G*_*r*_ are far more favorable for the incomplete reduction of NO_3_^–^, over 30 kJ (mol e^–^)^–1^ at all temperatures.

Natural settings that could host the manganese redox reactions noted in this study are widespread. Sediments in general serve as plausible locations for comproportionation, disproportionation and Mn^2+^ oxidation reactions since they can contain coexisting Mn-oxides in particle form and aqueous Mn^2+^ (Luther et al., 1997) and ligand-bound Mn^3+^ in pore fluids (Madison et al., 2011, 2013; see Table 1), in addition to multiple oxidants such as oxygen, nitrate (see below), nitrite, and iron hydroxides (Schulz and Zabel, 2006). Disproportionation of Mn^3+^ could also occur in the redox-stratified water bodies where it has been found, such as the Chesapeake Bay (Oldham et al., 2015), the St. Lawrence Estuary (Oldham et al., 2017b), the Black Sea (Trouwborst et al., 2006) and even in oxic portions of the water column (Oldham et al., 2017a). It should be noted that the energetics of reactions involving aqueous ligand-bound Mn(III) species will vary depending on the bond strength between Mn and the ligand, and therefore the identity of the ligand. Comproportionation reactions could occur in nearly any setting where Mn-oxides and appreciable aqueous Mn^2+^ coexist at neutral to high pH. As noted in the introduction, iron-manganese nodules on the seafloor, which are ubiquitous (Orcutt et al., 2020) could be one such location, especially according to the model described by Kato et al. (2019).

In addition to the seafloor and sediments, all of the Mn-based metabolisms considered in this study could be supported in aquifers throughout the world given their relatively large concentrations of aqueous Mn (see Table 1) and varying oxidation states. For instance, less than half of groundwater in the United States is considered to be oxic (DeSimone et al., 2015). Add in the fact that roughly one-third of United States ground water has a pH > 7.5 (DeSimone et al., 2015), and the thermodynamic stage is set for Mn-based catabolisms. It is especially enlightening to note that the inoculum used to demonstrate the first and only example of a microorganism catalyzing the oxidation of Mn^2+^ with O_2_ to gain energy was unsterilized municipal drinking water from Pasadena, California, which is typically a mixture of aquifer and surface water sources (Yu and Leadbetter, 2020).

If microorganisms are to gain energy from the manganese reactions considered in this study, they must be able to catalyze these reactions before abiotic processes consume the reactants, even though this is no guarantee that they will reap the energetic rewards. For instance, microorganisms have been shown to oxidize Mn^2+^ up to five orders of magnitude faster than abiotic oxidation (Tebo et al., 2004) and they are thought to dominate Mn^2+^ oxidation in most aquatic settings (Tebo et al., 2004, 2005). Despite the ubiquity of microbial Mn^2+^ oxidation, and the fact that Reaction 5 (O_2_ reduction) is exergonic above pH ∼4 (Figure 4A), it was only recently shown that a microorganism was able to use the energy liberated by this process (Yu and Leadbetter, 2020). The calculations summarized in Figures 4B–E show that it is thermodynamically possible that other electron acceptors are capable of oxidizing Mn^2+^, particularly NO_2_^–^ and NO_3_^–^, over a broad range of conditions that can be found in marine settings. In fact, laboratory incubations have demonstrated the oxidation of Mn^2+^ by NO_3_^–^ (forming N_2_, Reaction 8) in sediments taken from continental margins (Luther et al., 1997) and Long Island Sound (Hulth et al., 1999), a process that had been previously proposed to occur (Aller et al., 1990; Schulz et al., 1994; Murray et al., 1995). Hulth et al. (1999) report a Gibbs energy for this reaction of −6.11 kJ (mol e^–^)^–1^ at pH 7 and −8.93 kJ (mol e^–^)^–1^ at pH 8. By comparison, we determined values of −10.3 kJ (mol e^–^)^–1^ and −14.9 kJ (mol e^–^)^–1^ at these values of pH. The differences are due to the differing activities of the aqueous species, particularly the concentration of N_2_ used in the reactions quotient, Equation 2: Hulth et al. (1999) used atmospheric N_2_ partial pressure (0.781 atm) and we used an activity of 10^–4^).

A number of studies have reported abiotic manganese disproportionation and comproportionation reactions in laboratory experiments. Typically, these experiments involve exposing an Mn-oxide to Mn^2+^, and analyzing the resulting material for particular Mn phases. For instance, several authors report that comproportionation reactions, like Reaction 3, are responsible for the formation of MnOOH when Mn^2+^ is added to birnessite (δ-MnO_2_) (Tu et al., 1994; Elzinga, 2011; Zhao et al., 2016). Under similar experimental conditions, both Mn disproportionation and comproportionation have been reported (Elzinga and Kustka, 2015; Elzinga, 2016; Hinkle et al., 2016). The addition of complex organic substances to Mn^2+^ and Mn-oxide can lead to the formation of MnOOH and Mn_3_O_4_ phases (Wang et al., 2018), while the addition of bacterial spore coatings are thought to drive both comproportionation and disproportionation reactions (Bargar et al., 2005). *Bacillus* spores have also been shown to be associated with the formation of mixed (i.e., III/IV) Mn-oxides over a broad range of temperatures (0–80°C) and Mn^2+^ concentrations (<1 nM to >25 mM), using a variety of ionic strengths (1 M HEPES and seawater) (Mandernack et al., 1995). Spore coats from marine *Bacillus* species at pH 7.5 have been shown to oxidize Mn^2+^ to amorphous Mn-oxide that later recrystallized to hausmannite (Mann et al., 1988).

The rates of the comproportionation and disproportionation reactions noted above are difficult to discern because these reactions are typically inferred based on an analysis of the Mn phases at the conclusion of the experiments. However, most of the experiments took place over days or weeks, so microorganisms would likely be able to catalyze the inferred reactions faster than the abiotic reactions occur. This is certainly the case with abiotic Mn^2+^ oxidation, which is kinetically slow (Hinkle et al., 2016 and references therein). On the other side of the catalytic spectrum, Mn^3+^ disproportionates rapidly abiotically, though when it complexes with organics and pyrophosphate, it remains stable (Kostka et al., 1995; Klewicki and Morgan, 1998; Luther et al., 1999; Parker et al., 2004) for an undetermined amount of time. Mn-oxides have been shown to catalyze the disproportionation of Mn(III)-phosphate complexes at high and low pH (Qian et al., 2019). It should also be noted that bacteriogenic MnO_2_, which is riddled with crystallographic defects filled with other cations, is quickly reduced to Mn^2+^ in the presence of ligands or sunlight (Spiro et al., 2010). Furthermore, as the amount of energy available from these redox reactions decreases, the rate of microbial catalysis can drop below detection levels (Jin and Bethke, 2003; LaRowe et al., 2012), perhaps even fading to 0 despite a remaining energetic drive (i.e., Δ*G*_*r*_ < 0) (Schink, 1997; Curtis, 2003; Jin and Bethke, 2003; Hoehler, 2004; LaRowe and Van Cappellen, 2011). Consequently, any search for novel Mn-based metabolisms should be focused on the combinations of temperature and composition that yield the most negative value of Δ*G*_*r*_: neutral to basic pH for comproportionation reactions as well as Mn^2+^ oxidation by NO_2_^–^, NO_3_^–^, and FeOOH; and nearly any conditions for Mn(III) disproportionation.

## Data Availability Statement

The original contributions presented in the study are included in the article/Supplementary Material, further inquiries can be directed to the corresponding author/s.

## Author Contributions

DL and JA conceived of the study. DL carried out the calculations and wrote the manuscript with input from JA. HC contributed to the display items and the bibliography. All authors contributed to the article and approved the submitted version.

## Conflict of Interest

The authors declare that the research was conducted in the absence of any commercial or financial relationships that could be construed as a potential conflict of interest.
