# Supplementary material for: The Energetic Potential for Undiscovered Manganese Metabolisms in Nature
Source: Front Microbiol. 2021 Jun 9;12:636145. doi: 10.3389/fmicb.2021.636145 (PMC8220133; doi:10.3389/fmicb.2021.636145)
Supplement: Supplementary file 1 [file Data_Sheet_1.DOCX]

**Supplementary Section**

Here, we use Reaction 5 in Table 3 to illustrative how the thermodynamic calculations carried out in this study were done:

2Mn^2+^ + O_2(aq)_ +2H_2_O $\to$ 2MnO_2_ + 4H^+^ . (S1)

As we note in the Methods section, values of overall Gibbs energies at the temperature, pressure and compositional conditions of interest, $\Delta G_{r}$, are calculated using

$\Delta G_{r}=\Delta G_{r}^{0}+RT \ln Q_{r}$ (S2)

where $\Delta G_{r}^{0}$ and *Q_r_* refer to the standard state Gibbs energy and reaction quotient of the indicated reaction, respectively, *R* represents the gas constant (8.314 J K^-1^ mol^-1^), and *T* denotes temperature in Kelvin. Below, we separately discuss how the two terms on the right-hand side of Eq. (S2) were calculated for Reaction (S1).

S.1 $\Delta G_{r}^{0}$ – standard state Gibbs energies of reaction

Values of standard state Gibbs energies of reaction, $\Delta G_{r}^{0}$, are calculated from

$$\Delta G_{r}^{0}=\sum_{i} \nu_{i}\Delta G_{\mathrm{products}}^{0}-\sum_{i} \nu_{i}\Delta G_{\mathrm{reactants}}^{0}$$

(S3)

where *ν_i_* stands for the respective stoichiometric coefficient of the *i*th product and reactant in a reaction and the $\Delta G_{\mathrm{products}}^{0}$ and $\Delta G_{\mathrm{reactants}}^{0}$ terms refer to the standard state Gibbs energy of formation for the products and reactants appearing in a chemical reaction, respectively. For Reaction (S1), $\Delta G_{r}^{0}$ is expressed as

$\Delta G_{r}^{0}=2\Delta G_{\mathrm{MnO}_{2}}^{0}+4\Delta G_{H^{+}}^{0}-2\Delta G_{\mathrm{Mn}^{2+}}^{0}-\Delta G_{O_{2\left( aq \right)}}^{0}-2\Delta G_{H_{2}O}^{0}$ (S4)

where each of the Gibbs energy symbols on the right side of Eq. (S4) refer to the standard state Gibbs energies of formation from the elements,$\Delta G_{f}^{0},$ of the subscripted species at a given pressure and temperature. When thermodynamic data are reproduced in textbooks, these are what are generally reported – at the reference pressure and temperature of 1 bar and 25^o^C. Values of $\Delta G_{f}^{0}$ at temperatures and pressures other than these reference conditions are calculated using:

$$G_{P,T}^{0}-G_{P_{r},T_{r}}^{0}=-S_{P_{r},T_{r}}^{0}\left( T-T_{r} \right)+\int_{T_{r}}^{T} C_{P_{r}}^{0}dT-T\int_{T_{r}}^{T} C_{P_{r}}^{0}d\ln T+\int_{P_{r}}^{P} V^{0}dP$$

(S5)

Where $G_{P,T}^{0}$ denotes the standard state Gibbs energy of a species at any temperature and pressure, $G_{P_{r},T_{r}}^{0}$stands for the standard state Gibbs energy of a species at the *reference* temperature of 25^o^C and pressure of 1 bar, refers to the standard (third-law) entropy of the species at the reference pressure and temperature, *T_r_* denotes the reference temperature, $C_{P_{r}}^{0}$ stands for the isobaric molal heat capacity at the reference pressure and $V^{0}$ designates the standard molal volume. Values of $C_{P_{r}}^{0}$ and $V^{0}$ can vary as function of temperature, and $V^{0}$ can also vary with pressure. Consequently, the $C_{P_{r}}^{0}$ and $V^{0}$ terms in Eq. (S5) can be represented by functions that capture their variability as a function of temperature (and pressure for volume). For example, $C_{P_{r}}^{0}$ for solid mineral phases as a function of temperature is often represented by the Maier-Kelley equation (Maier and Kelley, 1932),

$$C_{P_{r}}^{0}=a+bT+cT^{-2}$$

(S6)

where the *a*, *b* and *c* parameters are species-dependent coefficients determined by regressing heat capacity data as a function of temperature with Eq. S6 (the thermodynamic data shown in Table 2 contain values of these parameters for some of the Mn-bearing minerals). Substitution of Eq. (S6) into Eq. (S5) and integrating the heat capacity terms leads to

$${\Delta G}_{P,T}^{0}-{\Delta G}_{P_{r},T_{r}}^{0}=-S_{P_{r},T_{r}}^{0}\left( T-T_{r} \right)+a\left( T-T_{r}-T\ln\left( \frac{T}{T_{r}} \right) \right)-\left( \frac{\left( c-bT_{r}^{2}T \right)\left( T-T_{r} \right)^{2}}{2T_{r}^{2}T} \right)+\int_{P_{r}}^{P} V^{0}dP$$

(S7).

This type of formulation is used to calculate how standard state Gibbs energies for many solids, liquids and gases differ from those at the reference conditions as a function of temperature and pressure (other formulations are used depending on the temperature and pressure range considered as well as the types of chemical substances; for this study, values of $V^{0}$ for solids are assumed to not change with pressure). The analogous expression for aqueous compounds is far more complex than what is shown in Eq. (S7), and is not shown here. Briefly, we used the revised HKF equations of state (Helgeson et al., 1981; Tanger and Helgeson, 1988; Shock et al., 1992) to calculate how the standard state Gibbs energy of aqueous species change as a function of temperature and pressure, relative to the reference temperature and pressure. These equations take into account how aqueous species, such as Mn^2+^, interact with a solvent, water. That is, the revised HKF equations of state partition thermodynamic functions into structural (non-solvation) and solvation terms. The solvation contributions are expressed in terms of Born transfer theory (see Shock et al., 1992), while the non-solvation components are determined from regressions of thermodynamic data such as heat capacity, volumes and isothermal compressibilities (see LaRowe and Helgeson (2006) for the revised-HKF equations for neutral and charged aqueous species).

The values of ${\Delta G}_{P,T}^{0}$ for the chemical species in Reaction (1) are given as function of temperature and pressure in Table S1. It can be seen here that values of ${\Delta G}_{P,T}^{0}$ for H^+^ are all 0. This is what is known as the hydrogen ion convention – all thermodynamic properties of H^+^ are taken to be 0 at all temperatures and pressures (see Cox et al. (1989) and Johnson et al. (1992)). The values of $\Delta G_{P,T}^{0}$ for H_2_O are calculated using well-established formulations (Levelt Sengers et al., 1983; Haar et al., 1984).

Table S1.

| $\Delta G_{f}^{0}$ (*T*, *P*) | 0.01^o^C, 1 bar | 25^o^C, 1 bar | 50^o^C, 1 bar | 75^o^C, 1 bar | 100^o^C, 1.0133 bar |
| --- | --- | --- | --- | --- | --- |
| Mn^2+^ | -232.2 | -230.5 | -228.8 | -227.1 | -225.4 |
| O_2(aq)_ | 18.99 | 16.54 | 13.59 | 10.25 | 6.56 |
| H_2_O | -235.5 | -237.2 | -239.0 | -241.0 | -243.1 |
| MnO_2 pyrolusite_ | -464.8 | -466.1 | -467.5 | -469.0 | -470.6 |
| H^+^ | 0 | 0 | 0 | 0 | 0 |
| $\Delta G_{r}^{0}$ | -13.23 | -13.30 | -12.86 | -11.99 | -10.76 |

Values of the standard state Gibbs energies of formation, $\Delta G_{f}^{0}$, for the chemical species in Reaction (S1) and the standard state Gibbs energy of reaction, $\Delta G_{r}^{0}$, for Reaction (S1) as a function of temperature and pressure. The units for all of these values are kJ mol^-1^. The pressure at 100^o^C is slightly higher than that at the other temperatures such that water remains liquid.

S.2 *Qr* – the reaction quotient term

Values of the reaction quotient, *Q_r_*, in Eq. (S2) are calculated with

$Q_{r}=\prod_{i} a_{i}^{\nu_{i}}$ , (S8)

where *a_i_* stands for the activity of the *i*th species and *v_i_* corresponds to the stoichiometric coefficient of the *i*th species in the reaction of interest. Activities are the thermodynamic measure of concentration. Under some circumstances, concentrations and activities are equivalent or nearly so. They differ as the composition of the environment stray further from standard state conditions, which specify a standard state of composition and aggregation, not a temperature and pressure. We specified activities of all substances appearing as reactants and products in this study, not concentrations. Therefore, we did not have to determine values of activity coefficients to convert concentrations to activities. For representative values of activity coefficients, see Amend and LaRowe (2019).

The specific form of *Q_r_* for Reaction (S1) is given by

$$Q_{r}=\frac{a_{\mathrm{MnO}_{2}}^{2}a_{H^{+}}^{4}}{a_{\mathrm{Mn}^{2+}}^{2}a_{O_{2(aq)}}a_{H_{2}O}^{2}}$$

(S9)

The activities of pure solids and liquids are taken to be 1. Therefore, values of *Q_r_* for Reaction (S1) are determined by the activities of H^+^, Mn^2+^ and O_2_. Furthermore, it can clearly be seen that for equivalent variations in activities of these three species, changes in pH ($\mathrm{pH}=-\log a_{H^{+}}$) have the largest impact on values of *Q_r_* since $a_{H^{+}}$ is raised to the fourth power. This is why we focused on the role of pH influencing values of $\Delta G_{r}$ in this study. Values of *Q_r_* and *RT* ln *Q_r_* for Reaction (S1) are given in Table S2 as a function of pH at 50^o^C.

Table S2

| pH | log *Q_r_* | *RT* ln *Q_r_* |
| --- | --- | --- |
| 1 | 9 | 55.7 |
| 3 | 7 | 43.3 |
| 5 | 5 | 30.9 |
| 7 | 3 | 18.6 |
| 9 | 1 | 6.18 |
| 11 | -1 | -6.18 |
| 13 | -3 | -18.6 |

Values of the logarithm of the reaction quotient *Q_r_* and *RT* ln *Q_r_* for Reaction S1 as a function of pH at 50^o^C, where *R* represents the gas constant (8.314 J K^-1^ mol^-1^), and *T* denotes temperature in Kelvin. The values of *Q_r_* are calculated using Eq. (S9). The activities of H_2_O and MnO_2_ are taken to be 1 and those for Mn^2+^ and O_2,(aq)_ are 10^-6^ and 10^-4^, respectively. Values of $a_{H^{+}}$ are related to pH by: $a_{H^{+}}={10}^{-\mathrm{pH}}$. The units for *RT* ln *Q_r_* are kJ mol^-1^ while pH and *Q_r_* are dimensionless.

**Supplementary Section References**

Amend, J.P. and LaRowe, D.E. (2019) Minireview: demystifying microbial reaction energetics. *Environmental Microbiology* **21**, 3539-3547.

Cox, J.D., Wagman, D.D. and Medvedev, V.A. (1989) *CODATA Key Values for Thermodynamics*. Hemisphere, New York.

Haar, L., Gallagher, J.S. and Kell, G.S. (1984) *NBS/NRC steam tables*. Hemisphere, Washington, D.C.

Helgeson, H.C., Kirkham, D.H. and Flowers, G.C. (1981) Theoretical prediction of thermodynamic behavior of aqueous electrolytes at high pressures and temperatures: 4. Calculation of activity coefficients, osmotic coefficients, and apparent molal and standard and relative partial molal properties to 600^o^C and 5 kb. *Amer. J. Sci.* **281**, 1249 - 1516.

Johnson, J.W., Oelkers, E.H. and Helgeson, H.C. (1992) SUPCRT92 - A software package for calculating the standard molal thermodynamic properties of minerals, gases, aqueous species, and reactions from 1 bar to 5000 bar and 0^o^C to 1000^o^C. *Comput. Geosci.* **18**, 899 - 947.

LaRowe, D.E. and Helgeson, H.C. (2006) Biomolecules in hydrothermal systems: Calculation of the standard molal thermodynamic properties of nucleic-acid bases, nucleosides, and nucleotides at elevated temperatures and pressures. *Geochim. Cosmochim. Acta* **70**, 4680-4724.

Levelt Sengers, J.M.H., Kamgar-Parsi, B., Balfour, F.W. and Sengers, J.V. (1983) Thermodynamic properties of steam in the critical region. *J. Phys. Chem. Ref. Data* **12**, 1-28.

Maier, C.G. and Kelley, K.K. (1932) An equation for the representation of high-temperature heat content data. *J. Amer. Chem. Soc.* **54**, 3243-3246.

Shock, E.L., Oelkers, E., Johnson, J., Sverjensky, D. and Helgeson, H.C. (1992) Calculation of the thermodynamic properties of aqueous species at high pressures and temperatures - Effective electrostatic radii, dissociation constants and standard partial molal properties to 1000^o^C and 5 kbar. *J. Chem. Soc. Faraday Trans.* **88**, 803 - 826.

Tanger, J.C. and Helgeson, H.C. (1988) Calculation of the thermodynamic and transport properties of aqueous species at high pressures and temperatures - Revised equations of state for the standard partial molal properties of ions and electrolytes. *Amer. J. Sci.* **288**, 19 - 98.
